# Does hysteroscopy worsen prognosis in women with type II endometrial carcinoma?

**DOI:** 10.1371/journal.pone.0174226

**Published:** 2017-03-23

**Authors:** Jiao Chen, Leslie H. Clark, Wei-Min Kong, Zhen Yan, Chao Han, Hui Zhao, Ting-Ting Liu, Tong-Qing Zhang, Dan Song, Si-Meng Jiao, Chunxiao Zhou

**Affiliations:** 1 Department of Gynecological Oncology, Beijing Obstetrics and Gynecology Hospital, Capital Medical University, Beijing, China; 2 Division of Gynecologic Oncology, Department of Obstetrics and Gynecology, University of North Carolina at Chapel Hill, Chapel Hill, North Carolina, United States of America; 3 Lineberger Comprehensive Cancer Center, University of North Carolina at Chapel Hill, North Carolina, United States of America; Taipei Medical University, TAIWAN

## Abstract

**Background:**

Prior studies evaluating the impact of hysteroscopy on outcomes in endometrial cancer have predominantly evaluated type I tumors. We sought to evaluate whether hysteroscopy worsens prognosis in type II endometrial cancer.

**Methods:**

A retrospective cohort analysis of 140 patients from two institutions with type II endometrial cancer was performed. Women who underwent either diagnostic hysteroscopy (HSC) or dilation and curettage (D&C) for cancer diagnosis from June 2001 until June 2010 were included. The clinical and pathologic characteristics, including peritoneal cytology results were reviewed. The primary endpoint was disease-specific survival (DSS). The exposure of interest was hysteroscopy. Survival curves were projected using the Kaplan-Meier method and compared using the log-rank test.

**Results:**

There was no difference in age, histology, stage, depth of myometrial invasion, adnexal involvement, or nodal metastasis between HSC and D&C patients. Positive cytology was found in 16/54 (30%) patients following HSC and in 10/86 (12%) following D&C (p = 0.008). Fourteen patients with stage I and II disease had positive peritoneal cytology, with 11/40 (27.5%) patients in the HSC group and 3/59 (5%) patients in the D&C group(p = 0.002). Median DSS was clinically different for the HSC and D&C groups, but statistical significance was not reached (53 versus 63.5 months, p = 0.34). For stage I and II patients, 18/99 (18%) were dead of EC, with a median DSS of 60 months for HSC and 71 months for D&C (p = 0.82). Overall 46 (33%) patients developed a recurrence, with 18/54 (33%) in the HSC group compared to 28/86 (32%) in the D&C group (p = 0.92). There was no difference in recurrence location between groups.

**Conclusions:**

Diagnostic hysteroscopy significantly increased the rate of positive peritoneal cytology at the time of surgical staging in this cohort of patients with type II EC. However, we were unable to detect a difference in prognosis as measured by DSS.

## Introduction

Endometrial cancer (EC) is the most common malignancy of the female genital system, and can be classified into type I and type II tumors[1]. Type I tumors are of endometrioid histology and are the most common type of EC. These tumors are associated with estrogen dependence and favorable prognosis [2–4]. Type II tumors include a variety of histologies such as serous and clear cell carcinomas. These tumors are associated with poor clinical outcomes [3–5]. More recently, carcinosarcoma has also been included in type II tumors as it is considered a metaplastic carcinoma by the 2010 National Comprehensive Cancer Network (NCCN) panel [6]. Compared with type I EC, type II EC behaves more aggressively with a higher incidence of extrauterine disease and increased propensity for recurrence[7,8]. The 5-year survival rate for type II EC is reported to be between 30% and 60% [9–11].

Hysteroscopy (HSC) is a procedure that is often used to assist in the diagnosis of EC. The accuracy of hysteroscopically-guided curettage was shown to be higher than D&C alone in a meta-analysis and is preferred by some providers [11]. However, due to the use of distention media and increased intrauterine pressure, there is a theoretical concern that HSC may facilitate the dissemination of malignant cells into peritoneal cavity though the fallopian tubes. Most retrospective studies have found that despite the higher prevalence of positive peritoneal cytology following HSC, there is no evidence to suggest that HSC is associated with increased mortality or worsened prognosis in women with EC [12–17]. However, to date, all studies evaluating the risk of HSC in EC prognosis have predominantly included patients with type I EC. With no studies specifically addressing type II EC, the effect of peritoneal dissemination of malignant cells in this subset of EC remains less clear. We hypothesized that peritoneal dissemination of malignant cells following HSC in type II EC, due to its more aggressive nature, would negatively impact the prognosis of these patients. Thus, we conducted a retrospective cohort study among patients with type II EC to evaluate the effect of HSC on survival.

## Materials and methods

### Study design, settings, participants

Following institutional review board approval at both institutions, all cases of type II EC treated at Beijing Obstetrics and Gynecology Hospital in Beijing, China and the University of North Carolina Women’s Hospital in Chapel Hill, North Carolina, USA from June 2001 to June 2010 were identified. Inclusion criteria were a confirmed pathologic diagnosis of type II EC and available peritoneal cytologic results. Patients who did not undergo peritoneal cytology were excluded from this study. Additional exclusion criteria included (1) endometrioid histology or non-epithelial histology, (2) synchronous malignancy, and (3) evidence of lymphatic or distant metastasis at the time of diagnosis, and (4) diagnosis via method other than D&C or HSC. Overall, 165 histologically confirmed cases of type II EC were identified. Thirteen cases were excluded due to lack of peritoneal cytology results, 5 cases for preoperative evidence of lymphatic or distant metastasis and 4 cases based on not undergoing HSC or D&C for diagnosis. Overall, 140 cases met the criteria for evaluation and were included in this retrospective cohort analysis. STROBE guidelines were followed in the design and reporting of this study [18].

### Data sources and variables

Demographic and clinical information were abstracted for each patient using medical records. For study purposes all patients were staged using the 2009 International Federation of Gynecology and Obstetrics (FIGO) staging system for EC [19]. The following demographic and clinical data were obtained: age, preoperative diagnosis, diagnostic procedure performed (D&C or HSC), FIGO 2009 stage, postoperative histologic type, depth of myometrial invasion, cervical stromal involvement, lymph vascular space invasion, adnexal and lymph nodes metastasis, adjuvant therapy received, disease recurrence date and location, and date and cause of death or last follow up.

The exposure of interest was HSC. Referral records and operative reports were obtained to determine if a patient underwent HSC with biopsy/D&C (n = 54) or D&C alone (n = 86) for diagnosis. Patients who underwent only office endometrial biopsy were excluded from this analysis. The D&C and HSC could be performed at either of the two study institutions or at an outside institution prior to referral. D&C was performed using traditional curettage biopsy. HSC was performed with or without anesthesia as per institutional practice. The uterine cavity was then distended with either 0.9% sodium saline or 5% glucose solution to a pressure range of 80–110mmHg. Following HSC, either traditional curettage or hysteroscopic-directed biopsy was performed. In the HSC group, 51 patients (94%) performed HSC followed by curettage, and 3 patients (6%) underwent HSC with directed biopsy.

Following the diagnostic procedure, surgical staging was performed in all patients. Almost all patients had pelvic ultrasonography or pelvic and abdominal CT or MRI examination before surgery, except patients diagnosed after surgery. Presumed early stage patients underwent planned complete surgical staging with total hysterectomy and bilateral salpingo-oophorectomy with peritoneal washings in all patients. Complete pelvic and para-aortic lymph node dissections were performed as feasible. Both open and minimally invasive surgeries were included. All patients with evidence of advanced stage at the time of surgery underwent a tumor cytoreductive surgery, however, patients with evidence of advanced stage disease at the time of diagnosis were excluded from this study. Ascites or peritoneal washings were taken in all patients included in this study. Experienced gynecologic cytopathologists and gynecologic pathologists at the corresponding institution reviewed all cytologic and pathologic samples.

Adjuvant treatment with either radiotherapy and/or adjuvant chemotherapy were administered per institutional practices for incomplete tumor resections, lymph node metastases, deep myometrial invasion, adnexal metastases, and extensive lymphovascular space invasion. Adjuvant radiotherapy included the use of external beam pelvic radiation (4000~ 4500cGy) and vaginal brachytherapy. When adjuvant chemotherapy was administered the regimens used included platinum/paclitaxel (carboplatin AUC 4–6 or cisplatin 60mg/m^2^, d1; paclitaxel 175 mg/m^2^, d1, q 21 days) and PAC (carboplatin AUC 4–6 or cisplatin 60mg/m^2^, d1; epirubicin 50mg/m^2^, d1; cyclophosphamide 500mg/m^2^, q 21 days). The courses of chemotherapy ranged from 2 to 6.

Patients were followed from the time of surgery until June 2015 for the purposes of this study. Patients were followed up at 3-month intervals for the first 2 years, at 6-month intervals for the next 3 years and, thereafter, once a year from the fifth year on. The primary outcome of interest was disease-specific survival (DSS). DSS was defined as the interval from the date of surgery to death from disease. Patients were censored at the time of their last follow up. Secondary outcomes included progression-free survival (PFS), stage at diagnosis, presence of positive peritoneal cytology at the time of staging surgery, and recurrence patterns. For patients with stage III and IV disease, the beginning of the disease free interval was defined as no residual tumor on imaging, absence of clinical symptoms, and normalization of tumor markers if elevated prior to therapy. PFS was defined as the time interval from surgery to the first evidence of recurrence (using clinical exam findings or radiologic findings) or death from any cause.

### Statistical methods

Statistical analyses were performed using SPSS (version 17.0) statistical software. Continuous variables were compared using Student’s t test. Categorical variables were compared using *x*^*2*^ test. PFS and DSS were estimated with the Kaplan-Meier method and survival curves were compared by log-rank test. A *p* value of less than 0.05 was considered statistically significant.

## Results

### Participants’ demographic and clinical characteristics

The average age at diagnosis was 61.2±9.5 years. One hundred and twenty-three patients (88%) were postmenopausal. Sixty patients (43%) had serous adenocarcinoma, 37 (26%) had clear cell carcinoma, 31 (22%) had carcinosarcoma and 12(9%) had mixed histology. All patients underwent total hysterectomy and bilateral salpingo-oophorectomy with 132 (94%) patients undergoing pelvic lymph node dissection and 91(65%) also undergoing aortic lymph node dissection. Adjuvant therapy was administered to 111(79%) patients. Of those receiving adjuvant therapy, 15(11%) received adjuvant radiation only (XRT), 34(24%) received adjuvant chemotherapy (CT) only, and 62 (44%) received both XRT and CT. Overall, 76 (54%) were stage I, 23(16%) were stage II, 33 (24%) were stage III, and 8(6%) were stage IV.

Comparing the HSC group (n = 54) to the D&C only group (n = 86), there was no significant difference in age at diagnosis (61.4 vs. 61.1 years, p = 0.86), histology (46% serous vs. 41% serous, p = 0.65), stage at diagnosis (26% stage III/IV vs. 31%, p = 0.81), or receipt of adjuvant treatment (p = 0.22). There was also no difference in pathologic characteristics between groups with 39% of the HSC group and 38% in the D&C group had outer half myometrial invasion (p = 0.95). Approximately one-third of each group had lymphovascular space invasion. Fifteen percent of patients in the HSC group had lymph node metastasis versus 23% in the D&C group (p = 0.22). These findings are summarized in Table 1.

**Table 1:** Comparison of clinical and pathological characteristics.

	Hysteroscopy	D&C	P value
	n = 54	n = 86	
Age (*SD*, years)	61.4 (9.3)	61.1 (9.7)	0.86
Histology			0.65
Serous	25 (46)	35 (41)	
Clear cell	11 (20)	26 (30)	
Carcinosarcoma	13 (24)	18 (21)	
Other/Mixed	5 (9)	7 (8)	
Stage			0.81
I	30 (56)	46 (53)	
II	10 (19)	13 (15)	
III	12 (22)	21 (24)	
IV	2 (4)	6 (7)	
Treatment*			0.22
S	7 (13)	22 (26)	
S+XRT	6 (11)	9 (10)	
S+CT	17 (31)	17 (20)	
S+XRT+CT	24(44)	38 (44)	
Outer half myometrial invasion	21 (39)	33 (38)	0.95
Lymph node metastasis	8 (15)	20 (23)	0.22
Lymphovascular space invasion	18 (33)	26 (30)	0.70
Adnexal metastasis	5 (9)	14 (16)	0.24

Continuous variables are reported as mean (+/- standard deviation); categorical variables are reported as n(%).

* S = surgery, XRT = radiation, CT = chemotherapy.

### Peritoneal cytology

Positive pelvic washings were seen on cytology in 26 patients (25%) overall. A higher incidence of positive washings was noted in patients undergoing HSC compared to D&C (n = 16, 30% versus n = 10, 12%, p = 0.008). Fourteen patients with stage I and II had positive peritoneal cytology, with 11/40 (28%) of patients in HSC group and 3/59(5%) patients in D&C group (p = 0.002). While for stage III and IV patients, positive peritoneal cytology were found in 5/14 (36%) of patients in HSC group and 7/27(26%) in D&C group, the rate had no significance (p = 0.51).

### Survival and recurrence patterns

**Fig 1:**
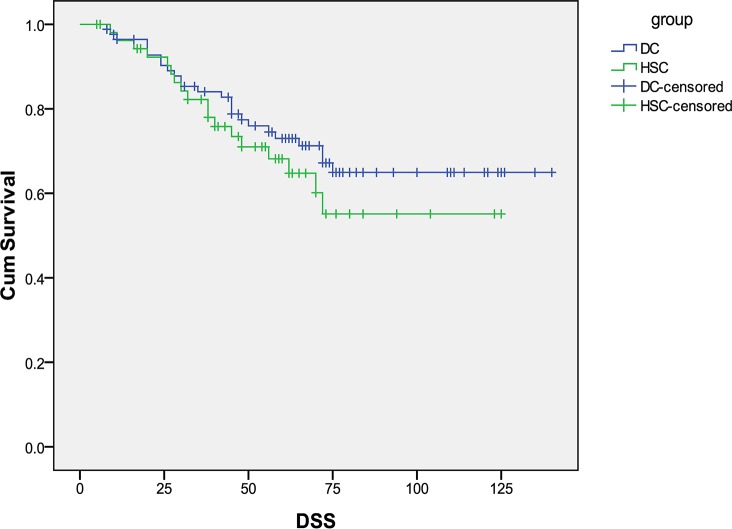
Disease-specific survival in patients with type II endometrial cancer diagnosed with hysteroscopy (HSC) versus D&C.

**Fig 2:**
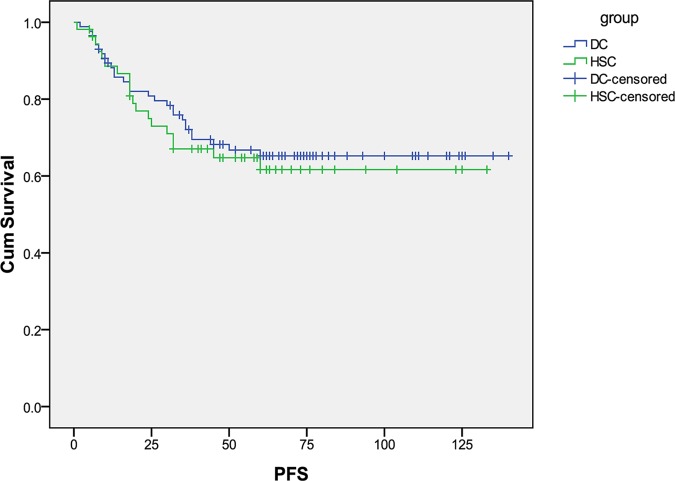
Progression-free survival in patients with type II endometrial cancer diagnosed with hysteroscopy (HSC) versus D&C.

The median overall follow-up time for this cohort was 66 months (range 6–140 months). For the two diagnostic subgroups, follow-up period ranged (median) from 8 to 140 (72) and 6 to 133 (61) months for D&C and HSC, respectively. At the time of analysis, 71 patients were alive without disease, 43 patients were dead of EC, and 9 cases were dead of other causes. Survival data was missing on 15 patients and these patients were censored at the time of last follow up. The DSS and PFS curves are shown in Fig 1 and Fig 2. There were no significant differences in DSS and PFS between D&C and HSC group for all stage patients. The median DSS was 53 months for HSC and 63.5 months for D&C (p = 0.34). The median PFS was 47.5 months for HSC and 60 months for D&C (p = 0.66).

**Fig 3:**
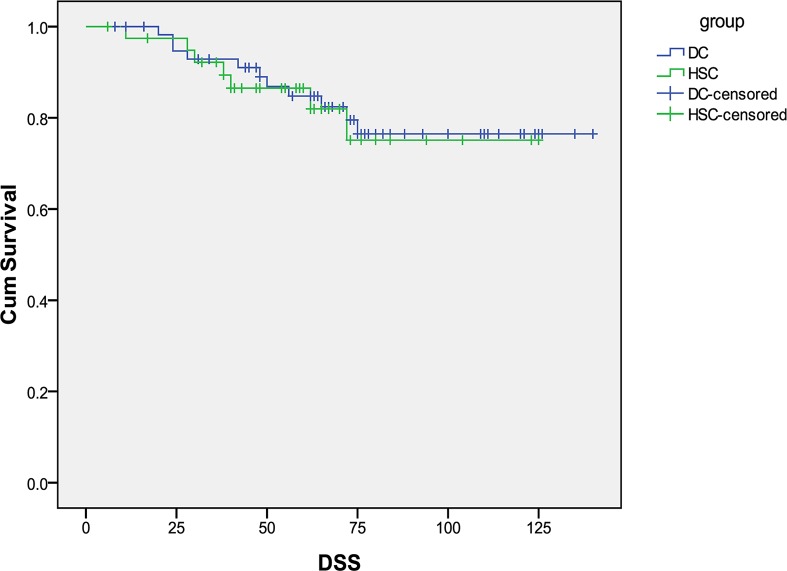
Disease-specific survival curves in patients with type II endometrial cancer for stage I and II diagnosed by HSC and D&C.

**Fig 4:**
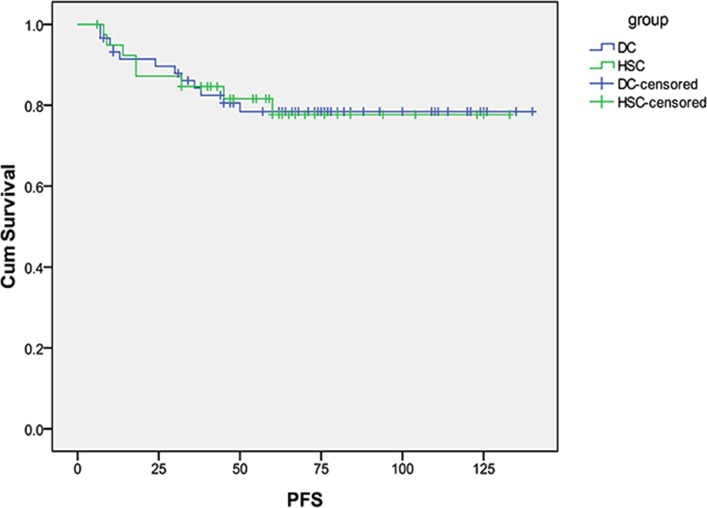
Progression free survival curves in patients with type II endometrial cancer for stage I and II diagnosed by HSC and D&C.

For stage I and II patients, 18/99 (18%) died of EC, The median DSS was 60 months for HSC and 71 months for D&C (p = 0.82). Fig 3 shows the DSS curves. The median PFS was 60 months for HSC and 68 months for D&C (p = 0.99). PFS is displayed in Fig 4.

Overall 46 patients (33%) experienced a disease recurrence. There were 18/54 (33%) patients with recurrence in the HSC group compared to 28/86 (32%) in the D&C group (p = 0.92). For stage I and II patients, 7/40 (18%) patients had a recurrence in the HSC group and 12/59 (20%) patients in the D&C group (p = 0.72). While for stage III and IV patients, recurrence developed in 11/14 (79%) patients in HSC group and 16/27 (60%) patients in D&C group (p = 0.22). Among the patients who recurred, 28 (61%) developed local recurrence, 25(54%) experienced distant metastasis, and 7 (15%) had both local and distant recurrence. The most common locations of distant metastasis were lung (11/25, 44%), but other sites included brain (4/25,16%), lymph nodes (9/25, 36%), and liver (1/25, 4%). There was no difference in site of recurrence between the two groups, both in early stage and advanced stage (Table 2).

**Table 2:** Recurrence patterns between groups.

	Stage I and II			Stage III and IV		
	n = 99			n = 41		
	Hysteroscopy	D&C	P value	Hysteroscopy	D&C	P value
	n = 40	n = 59		n = 14	n = 27	
Local regional recurrence	4(10)	9(15)	0.448	6(43)	9(33)	0.548
Vaginal cuff	2(50)	5(56)		2(33)	3(33)	
Pelvis	2(50)	4(44)		4(67)	6(67)	
Distant recurrence	4(10)	6(10)	1.000	7(50)	8(30)	0.199
Lung	2(50)	3(50)		2(29)	4(50)	
Brain	1(25)	0(0)		2(29)	1(12)	
Lymph nodes	1(25)	2(33)		3(43)	3(38)	
Liver	0(0)	1(17)		0(0)	0(0)	
Local and distant recurrence	1(3)	3(5)	0.904	2(14)	1(4)	0.217

Categorical variables are reported as n (%).

## Discussion

In the present study, we investigated whether HSC to aid in the diagnosis of EC lead to a worse prognosis in women with type II tumors. We found that HSC is associated with an increased rate of positive peritoneal cytology in patients with type II EC (30 versus 12%, p = 0.008), theoretically due to expulsion of malignant cells through the fallopian tubes after uterine distension. Despite this difference in peritoneal cytology, we were unable to detect a difference in prognosis with a median DSS of 53 versus 63.5 months in the HSC and D&C arms, respectively.

Regarding positive cytology, our study results are similar to prior studies that evaluated the effects of HSC on positive cytology in predominantly type I EC [20–25]. However, given the more aggressive nature of type II EC, this study adds to the literature by specifically evaluating the effect of HSC on type II tumors. Previously reported rates of positive cytology at the time of endometrial cancer staging have varied widely. Takac *et al* [20] reported a 12.5% rate of positive peritoneal cytology after HSC compared with 1.6% rate without HSC in 146 patients with EC. This cohort included predominantly endometrioid adenocarcinomas (86%), suggesting that diagnostic HSC increases the risk of positive peritoneal cytology. While some authors have not found evidence to support an increased rate of positive peritoneal cytology after HSC, two large meta-analyses have concluded that the rate of positive peritoneal cytology after HSC is significantly higher than after D&C alone[14,16]. In addition to focusing on type II EC, our study is unique in that the rate of positive cytology was higher than previous reported in the literature[12,21,22,24,26]. We hypothesize that the increased rate of positive cytology was likely due to the aggressive nature of type II EC with increased necrosis and cellular sloughing, as prior series have included relatively small numbers of type II tumors. Further, in order to decrease potential bias caused by advanced stage, the positive cytology rate in stage I and II tumors was compared between two groups with a significant difference seen.

While the finding of positive cytology is intriguing, the real question is whether this results in impaired clinical outcomes and worse survival. At present, studies on the effect of HSC on prognosis are predominately retrospective and focused on type I tumors. There has been no association between HSC and poor prognosis in studies to date [15,25,27–29]. One of the largest studies by Soucie *et al* evaluated 1972 patients with type I EC and found no association between advanced stage or mortality in patients undergoing HSC [15]. These authors concluded that HSC is a safe diagnostic tool for identifying EC. However, the prognostic effect of HSC has not been well described in the literature for type II tumors.

There is conflicting evidence regarding the prognostic significance of positive peritoneal cytology among patients with early stage EC, and the International Federation of Gynecology and Obstetrics (FIGO) staging criteria has eliminated cytology from surgical staging[30]. However, positive peritoneal cytology can add prognostic value, particularly in type II EC. *Han et al* found that positive peritoneal cytology is an independent prognostic factor for patients with non-endometrioid endometrial cancer [31]. Patients with positive peritoneal cytology were more frequently diagnosed with high-risk factors such as clear cell/ serous carcinoma and carcinosarcoma, and are at significant risk of lymph nodal metastasis and adverse prognosis[32]. In our cohort of type II tumors, there was no significant difference in survival with patients in the HSC group and D&C group. Tumor metastasis occurs via multiple potential mechanisms [33], including peritoneal dissemination, lymphatic spread and vascular dissemination. By limiting our analysis to presumed early stage patients and eliminating patients with known lymphatic and vascular spread on imaging we are able to focus on the role of peritoneal dissemination which could be increased following HSC. For stage I and II patients, we did see a clinically meaningful but statistically insignificant difference in survival with patients in the HSC group with a median DSS of 60 months compared to 71 months in the D&C group(p = 0.82).

One hypothesis to address the difference in rate of positive cytology but lack of survival difference seen in our study, as well as prior studies in type I tumors, is the inability of EC cells to survive when floating in the peritoneal cavity. Hirai *et al* [34] investigated the malignant potential of positive peritoneal cytology in EC and suggested that cancer cells found in the peritoneal cavity usually disappear within a short time. Perhaps, free floating tumor cells in the peritoneal cavity lack the microenvironment and nutrients needed for tumor growth resulting in loss of tumor activity. Additionally, we noted a high rate of adjuvant therapy and receipt of adjuvant radiotherapy and chemotherapy may affect the bioactivity of malignant cells in the peritoneal cavity before the invasion and metastasis can occur. However, other studies have shown that tumor cells disseminated at time of hysterectomy or HSC are biologically viable and have the ability to replicate *in vitro* [35]. In the absence of adjuvant treatment, peritoneal cell dissemination may result in adverse outcomes. Further supporting the importance of tubal dissemination of endometrial cancer are recent studies reporting that tubal ligation is associated with decreased stage of EC, reduced peritoneal metastasis, and improved mortality, particularly in serous and other high-risk histologic types[36–38].

Additionally, the current study is likely underpowered to detect a survival difference. Thus, while our study adds to the literature as the first to specifically evaluate the effect of HSC on type II EC, several limitations must be acknowledged. The main limitation is that a relatively small number of patients were included, given the rarity of type II EC. A post hoc power calculation suggests over 500 patients are needed to detect a survival difference. Another important limitation is the retrospective design of this study. There is a potential for selection bias with differing populations undergoing D&C versus HSC. Further, postoperative treatment was given without well-defined protocols for type II EC and may reflect institutional biases or practice patterns. Additionally, recent studies on molecular and pathological features, show that nearly 25% of endometrial cancers do not fall neatly into the traditional two tier system of endometrial cancer subtypes with significant molecular heterogeneity[39–41]. In this study we used the traditional Bokhman classification of EC (type I and II) while recognizing there may be a role for including grade 3 endometrioid carcinomas with traditional type II ECs based on clinical features and survival outcomes[33]. Further, other unmeasured risk factors or characteristics of the tumors may have influenced peritoneal dissemination and pelvic recurrence aside from HSC. Despite these limitations, we feel that our study provides more information on the effect of HSC on peritoneal cytology and outcomes in type II EC.

Our data for type II EC indicate that HSC significantly increases the rate of positive peritoneal cytology at the time of surgical staging in patients with type II EC. While we were unable to demonstrate a statistical difference in prognosis, we did note an interesting trend toward shorter median survival in the HSC group when evaluating patients with all stages of disease. We found no difference in recurrence rate or survival when limiting our analysis to stage I and II patients. Future studies are needed to evaluate the impact of HSC on prognosis in patients with type II EC and will likely require large collaborations to obtain the sample size needed for this relatively rare disease. Providers should understand a potential risk of peritoneal dissemination of tumor cells following HSC, particularly in type II tumors, however the effect on prognosis remains unclear.

## Supporting information

S1 Fig Primary data of parts of patients with type II EC. (XLSX)

## Abbreviations

HSC: Hysteroscopy
EC: endometrial cancer
D&C: dilation and curettage
DSS: disease-specific survival
NCCN: National Comprehensive Cancer Network
PFS: progression-free survival
CT: chemotherapy
XRT: radiation
